# Sensory Impairment and Beta-Amyloid Deposition in the Baltimore Longitudinal Study of Aging

**DOI:** 10.1093/geroni/igab046.1698

**Published:** 2021-12-17

**Authors:** Nicole Armstrong, Yurun Cai, Hang Wang, Jennifer Schrack, Yuri Agrawal, Eleanor Simonsick, Susan Resnick

**Affiliations:** 1 Warren Alpert Medical School of Brown University, Providence, Rhode Island, United States; 2 Johns Hopkins Bloomberg School of Public Health, Baltimore, Maryland, United States; 3 Johns Hopkins University, Baltimore, Maryland, United States; 4 Otolaryngology, Baltimore, Maryland, United States; 5 National Instute on Aging/NIH, Baltimore, Maryland, United States; 6 National Institute on Aging, Baltimore, Maryland, United States

## Abstract

Studies have demonstrated a link between sensory impairment and dementia risk, but little is known about the presence of beta-amyloid plaques in individuals with single and multisensory impairments. Sensory function (combinations of vision, hearing, vestibular function, and proprioception) and amyloid PET imaging were measured in 170 BLSA participants (age=78± 9 years; 53% women; 77% white; 28% amyloid positive) from 2012 to 2019. Log-binomial regression models were used to examine the prevalence ratios (PR) of amyloid positivity for individual sensory impairments and across categories of impairments. While crude associations indicate associations of vision impairment (PR=1.72, p=0.04) and impairments in all four senses (PR=2.38, p=0.03) with amyloid positivity, these associations were insignificant after adjusting for age, sex, race, and education. There were no other crude and adjusted associations. These results suggest sensory impairments may be related to dementia independent of AD pathology. Future studies with larger sample sizes are warranted.

